# Targeted treatment of autoimmune cytopenias in primary immunodeficiencies

**DOI:** 10.3389/fimmu.2022.911385

**Published:** 2022-08-16

**Authors:** Lucia Pacillo, Giuliana Giardino, Donato Amodio, Carmela Giancotta, Beatrice Rivalta, Gioacchino Andrea Rotulo, Emma Concetta Manno, Cristina Cifaldi, Giuseppe Palumbo, Claudio Pignata, Paolo Palma, Paolo Rossi, Andrea Finocchi, Caterina Cancrini

**Affiliations:** ^1^ Academic Department of Pediatrics (DPUO), Immune and Infectious Diseases Division, Research Unit of Primary Immunodeficiencies, IRCCS Bambino Gesù Children’s Hospital, Rome, Italy; ^2^ Department of Systems Medicine, University of Tor Vergata, Rome, Italy; ^3^ Pediatric Section, Department of Translational Medical Sciences, Federico II University, Naples, Italy; ^4^ Academic Department of Pediatrics (DPUO), Research Unit of Clinical Immunology and Vaccinology, IRCCS Bambino Gesù Children’s Hospital, Rome, Italy; ^5^ Department of Neuroscience, Rehabilitation, Ophthalmology, Genetics, Maternal and Child Health (DINOGMI), University of Genoa, Genoa, Italy; ^6^ Department of Onco Hematology, IRCCS Bambino Gesù Children’s Hospital, Rome, Italy

**Keywords:** primary immunodeficiency, inborn errors of immunity, autoimmune cytopenia, targeted therapy, immune dysregulation

## Abstract

Primary Immunodeficiencies (PID) are a group of rare congenital disorders of the immune system. Autoimmune cytopenia (AIC) represents the most common autoimmune manifestation in PID patients. Treatment of AIC in PID patients can be really challenging, since they are often chronic, relapsing and refractory to first line therapies, thus requiring a broad variety of alternative therapeutic options. Moreover, immunosuppression should be fine balanced considering the increased susceptibility to infections in these patients. Specific therapeutic guidelines for AIC in PID patients are lacking. Treatment choice should be guided by the underlying disease. The study of the pathogenic mechanisms involved in the genesis of AIC in PID and our growing ability to define the molecular underpinnings of immune dysregulation has paved the way for the development of novel targeted treatments. Ideally, targeted therapy is directed against an overexpressed or overactive gene product or substitutes a defective protein, restoring the impaired pathway. Actually, the molecular diagnosis or a specific drug is not always available. However, defining the category of PID or the immunological phenotype can help to choose a semi-targeted therapy directed towards the suspected pathogenic mechanism. In this review we overview all the therapeutic interventions available for AIC in PID patients, according to different immunologic targets. In particular, we focus on T and/or B cells targeting therapies. To support decision making in the future, prospective studies to define treatment response and predicting/stratifying biomarkers for patients with AIC and PID are needed.

## Introduction

Primary Immunodeficiencies (PID), nowadays referred to as Inborn Errors of Immunity (IEI) (1), are a group of rare congenital disorders of the immune system. They have been traditionally considered as conditions characterized by increased susceptibility to recurrent, atypical or severe infections, but with the progression of knowledge and long-term follow-up of these patients, the clinical spectrum has broadened especially to immune-dysregulatory manifestations including autoimmunity, hyperinflammation, lymphoproliferation and malignancies, that, sometimes, may represent the only or most prominent clinical feature (1).

The most common autoimmune manifestation in PID patients is represented by autoimmune cytopenias (AIC), that are rare, acquired conditions characterized by immune-mediated blood cells destruction. They include autoimmune haemolytic anaemia (AIHA), immune thrombocytopenia (ITP) and autoimmune neutropenia (AIN). In some cases, more than one AIC can occur at the same time or consequently, defining an Evans Syndrome (ES).

Fischer et al. (2) reported an incidence of immune-dysregulatory manifestations in 26% of the French national cohort of 2183 PID patients. Among 571 PID patients with immune-dysregulatory manifestations, AIC was observed in 31,4% (2). Particularly, AIHA, ES and ITP resulted respectively 830, 120 and 60 times more common in PID patients than in general population (2).

Moreover, pathogenic or potentially pathogenic variants have been detected in 65% of a cohort of 80 patients affected by Evans syndrome (3).

Actually, AIC are rare in patients with “typical” severe combined immune deficiency (SCID), while they are really common in patients with combined immune deficiency (CID), common variable immunodeficiency (CVID) or atypical SCID (AS) (2, 4). This could be explained since these last conditions are characterized by impaired but not abolished T cell function, that causes defective adaptive immune response against nonself antigens, as well as perturbs immune homeostasis by affecting central and peripheral mechanisms of immune tolerance (5).

Of note, AIC occurred in 84% of patients affected by Recombination Activating Gene (RAG) deficiency, presenting respectively AIHA in 60%, ITP in 36%, AIN in 33% and ES in 20% of all cases (6, 7). Among RAG deficiency patients, AIC are prevalent in those classified as affected by CID (8).

AIC pathogenesis is not well understood yet. Dissection of the mechanisms involved in immune dysregulation in RAG deficiency and in other forms of CID, in humans and in mouse models, has revealed distinct abnormalities in central and peripheral T- and B-cell tolerance as the key mechanisms (8). Understanding the pathophysiology of autoimmunity and hyperinflammation in these disorders is crucial for targeted therapeutic interventions.

Treatment of AIC in patients affected by PID can be really challenging, since in these patients AIC are often chronic, relapsing and refractory to first line treatments, requiring a broad variety of alternative therapeutic options. Moreover, immunosuppression should be fine balanced considering the increased susceptibility to infections in these patients. The goal would be to correct the underlying immune dysregulation minimizing nonspecific immunosuppression.

According to national and international guidelines (9–15) for the treatment of AIC, corticosteroids and high dose intravenous immunoglobulins (IVIg) represent the standard of care respectively for AIHA and ITP with a high response rate in general population. Around 80% of patients with AIC not associated with PID achieve remission with these therapeutic approaches (10, 11).

While first line treatment of autoimmune cytopenias is well standardized, there is no general consensus about second line therapies.

Given the toxicity concerns of non-selective immunosuppressants, especially in paediatric age, development of steroid-sparing therapeutic regimens has rapidly progressed in recent decades.

Second line therapies include Mycophenolate Mofetil (MMF), TPOR-Agonists (Eltrombopag, Romiplostin), mTOR inhibitors (Sirolimus, Everolimus) and anti-CD20 monoclonal antibody (Rituximab). Azathioprine, Cyclosporin, Vincristine, and other immunosuppressive or biological drugs, are used as third line treatments. Of note, many of these drugs of second and third line are off label for indication and/or for age. Finally, in some cases, splenectomy is considered in adults, but it is generally contraindicated in PID patients due to the risk of post-splenectomy sepsis.

The study of the pathogenic mechanisms involved in the genesis of AIC in PID and our growing ability to define the molecular underpinnings of immune dysregulation, thanks to the advent of new diagnostic techniques like Next-Generation Sequencing (NGS), has paved the way for the development of novel targeted treatments.

First, second- and third-line therapeutic options for ITP and AIHA are showed in **Table 1** (16).

**Table 1:** Therapeutic approaches for AICs. Adapted from SIC-REG.COM by Seidel (16), based on international guidelines (9–15).

AIHA, ES	ITP
FIRST LINE Prednisolone 2-5 mg/kg/d days 1-3, then 1-2 mg/kg/day, wean off after 4 wks>8wks	FIRST LINE OPTIONS IVIG 0.8-1 g/kg, repeatable If Rh+: anti-D (25) 50–75 μg/kg s.c. or i.v (not available in Europe) Predniso(lo)ne 4 mg/kg/d (max 200) in 3-4 doses (4 d) with no taper, or 1-2 mg/kg/d (max 80) for 1-2 wks. To taper in 3 wks.
SECOND LINE OPTIONS Prednisolone +MMF 1200 mg/m^2^/day If DNT ↑: Prednisolone + Sirolimus 1–2.8 mg/m2/day Notes: Wean off prednisolone after 4 weeks Wean off MMF after 6-12 months > 3-6 months If successful, keep dosage as low as possible If signs of CID: consider targeted therapy, HSCT Rituximab 375 mg/m2 once per week, 4 times especially in case of: chronic EBV infection, high CD19+frequency Methylprednisolone 10–30 mg/kg/d for 3 days Dexamethasone 5–10 mg/m^2^ > 4 d	SECOND LINE OPTIONS MMF max1200 mg/m^2^ day Wean off after 6-12 months > 3-6 months (if successful, keep dosage as low as possible) If DNT ↑: Sirolimus 2 to 2.5 mg/m2/day TPOR-Agonists: Eltrombopag 25-50 (75) mg/day (0.8-1.2 mg/kg <6y) orally Romiplostin (>18yrs or eltr. non responders) 100-250 ug/m (2 )week If signs of CID: consider targeted therapy, HSCT Rituximab (discussed as 2^nd^ or 3^rd^ line therapy) 375 mg/m2 once per week, 4 times especially in case of: chronic EBV infection, high CD19+ frequency
THIRD LINE OPTIONS Danazol, AZT, VCR, CY, CSA targeted treatments: Bortezomib, Daratumumab, Belimumab, Ibrutinib, Epratuzumab, Abatacept, PI3Kδ inhibitors, JAK Inhibitors, Anakinra, TNF inhibitors, Eculizumab, Plerixafor HSCT, GT adults: splenectomy (vaccinate before; consider OPSI prophylaxis)	THIRD LINE OPTIONS Rituximab, Danazol, AZT, VCR, Dapson targeted treatments: Bortezomib, Daratumumab, Belimumab, Abatacept, PI3Kδ inhibitors, Anakinra HSCT, GT adults: splenectomy (vaccinate before; consider OPSI prophylaxis)

AIHA, autoimmune haemolytic anaemia; AZT, azathioprine; CID, combined immunodeficiency; CY, cyclophosphamide; CYA, cyclosporine A; DNT, double negative T cells; ES, Evans Syndrome; GT, gene therapy; HSCT, hematopoietic stem cell transplant; IVIg, intravenous immunoglobulins; ITP, immune thrombocytopenia; MMF, mycophenolate mofetil; OPSI, overwhelming post-splenectomy infection; TPOR-agonists, thrombopoietin receptor agonists; VCR=vincristine.

In this review article we summarize the therapeutic approach to AIC in PID patients from well-known therapies to novel targeted treatments.

## First line therapeutic approaches

### Steroids

Corticosteroids immunosuppressive action is widely recognized and well-studied. They inhibit the synthesis of almost all cytokines and of several cell surface molecules required for immune function. In 1995 it was shown that glucocorticoids are potent inhibitors of nuclear factor kappa B (NF-kB), a known regulator of immune system and inflammation genes. This inhibition is mediated by induction of the IkBα inhibitory protein, which traps activated NF-kB in inactive cytoplasmic complexes (17).

Steroids represent nowadays the first-choice treatment in all cases of warm-type AIHA (9, 11, 12).

In mild to moderate cases of anaemia, standard treatment involves the use of oral prednisone at a dose of 2-5 mg/kg/day for day 1-3, then 1-2 mg/kg/day, to wean off after 4-8 weeks. In severe and symptomatic anaemia or in case of poor compliance to oral administration, intravenous methylprednisolone is used at a dose of 1-2 mg/kg every 6-8 hours for 1-3 days (11).

In severe cases, such as those with rapidly evolving and severe haemolysis, pulse high-dose intravenous methylprednisolone may be indicated (as a second line treatment) at a dose of 30 mg/kg/die, with a maximum dose of 1 g once daily for three days (12).

According to the most recent guidelines from the American Society of Haematology (13), steroids represent the standard of care also for ITP for minor-mild bleeding or in patients who are not responsive to IVIg.

Steroids are widely used for AIHA and ITP also in PID patients.

Nevertheless, the prolonged administration of steroids causes a large number of side effects, especially in paediatric age, like failure to thrive, alteration in bone development with osteoporosis and short stature, nonspecific immunosuppression with increased susceptibility to acute and chronic infections, behavioural and neuropsychiatric problems, gastric mucosa irritation, weight gain, cushingoid appearance, and so on. The risk of adverse effects is generally dose- and duration-dependent. This is the reason why many steroid-sparing therapies are considered, especially in PID patients, who often experience relapsing AIC.

### Intravenous immunoglobulins

IVIg immune regulatory function is not completely clear, but well recognized. Numerous targets for IVIg include: T-cells, cytokines, B-cells, complement and Fc-receptors (18). IVIg have been demonstrated to inactivate auto-reactive T-cells by competing for and interrupting their interaction with antigen presenting cells. The infused Ig bind to receptors on antigen presenting cells, increasing the expression of the inhibitory Fc-receptor and shortening the half-life of auto-reactive antibodies, stopping the autoimmune phenomenon (18).

IVIg represent the standard of care for ITP at a dosage of 0,80-1.00 g/kg in single dose, usually repeatable after 24 hours if a complete response is not observed and eventually repeatable after one month in cases of relapses (10, 13–15).

They are used as first line therapy for ITP also in PID patients, especially in ALPS (Autoimmune Lymphoproliferative Syndrome) and CVID patients, showing the same efficacy as idiopathic ITP (19). Nevertheless, since the recurrent relapses, sometimes it is necessary a monthly treatment that could compromise patient’s quality of life. Moreover, PID patients become often refractory to IVIg requiring other therapeutic options.

## Targeted therapies

Several authors have widely discussed the interplay between autoimmunity and PIDs (20–24). However, specific therapeutic guidelines for AIC in PID patients are lacking and a general consensus is not established. The treatment choice should be guided by the underlying disease.

Ideally, targeted therapy is directed against an overexpressed or overactive gene product or substitutes a defective protein, restoring the impaired pathway; it can also act indirectly, enhancing a counter mechanism against the disease-causing defect. Although making a definitive diagnosis of the suspected underlying IEI should be assigned first priority, it could take months until the specific IEI is identified. Meanwhile, a semi-targeted treatment guided by immune phenotype can be initiated.

Herein we review targeted therapeutic interventions available for autoimmune cytopenias in PID patients, basing on different immunologic targets, summarized in **Table 2**.

**Table 2:** Therapeutic agents and their targets and clinical use for AIC in PIDs.

THERAPEUTIC AGENT	MOLECULAR TARGET	CELLULAR TARGET	CLINICAL USE
***MMF***	IMPDH	T and B cells	AICs, many (25, 26)
***Rituximab***	CD20	mature B cells	AICs, many (27–30)
***Bortezomib***	proteasome	plasma cells	AICs, MM (31–38)
***Daratumumab***	CD38	plasma cells and plasma blasts	refractory AICs, MM (39–43)
***Isatuximab***	CD38	Plasma cells and plasma blasts	refractory AICs, MM (44, 45)
***Belimumab***	BAFF	B cells	AIC in SLE (46, 47)
***Atacicept***	BAFF and APRIL	B cells	SLE (48)
***Ibrutinib***	BTK	B cells	AIC in ALL (49, 50)
***Epratuzumab***	CD22	B cells	SLE (51)
***Sirolimus/Everolimus***	mTOR	T cells	ALPS, CTLA4 def, APDS, others (52–55)
***Abatacept***	CTLA4	T cells	CTLA4 def (56, 57), LRBA def (58)
***Leniolisib***	p110delta	T cells	APDS1 (59)
***Seletalisib***	p110delta	T cells	APDS1 and APDS2 (60)
***Tofacitinib***	JAK1, JAK3	T cells and inflammasome	JAK/STAT diseases (61–64)
***Ruxolitinib***	JAK1, JAK2	T cells and inflammasome	SAVI, STAT1 GOF, STAT3 GOF (61, 62)
***Baricitinib***	JAK1, JAK2	T cells and inflammasome	JAK/STAT diseases (61–64)
***Filgotinib***	JAK1	T cells and inflammasome	JAK/STAT diseases (61–64)
***Decernotinib***	JAK3	T cells and inflammasome	JAK/STAT diseases (61–64)
***Anakinra***	IL-1R	T and B cells	WAS (65), MIS-C (66, 67)
***Etanercept***	TNF	macrophages	DADA2 (68)
***Infliximab***	TNF	macrophages	DADA2 (68)
***Adalimumab***	TNF	macrophages	DADA2 (68)
***Plexirafor***	CXCR4	neutrophils	WHIM (69, 70)
***Mavorixafor***	CXCR4	neutrophils	WHIM (71)
***Eculizumab***	C5	complement	Rescue therapy of refractory AIHA (72, 73)

AIC, autoimmune cytopenia; ALL, acute lymphatic leukaemia; ALPS, autoimmune lymphoproliferative syndrome; APDS, Activated PI3K-kinase Delta Syndrome; DADA2, deficiency od adenosine deaminase; MIS-C, multisystem inflammatory syndrome in children; MM, multiple myeloma; MMF, mycophenolate mophetile; SAVI, STING-associated vasculopathy with onset in infancy; SLE, systemic lupus erythematosus; TNF, tumor necrosis factor; WHIM, Warts, hypogammaglobulinemia, infections, and myelokathexis; WAS, Wiskott-Aldrich Syndrome.

### Targeting T and B cells

The best-studied steroid-sparing agent used in children with AIC, off-label both for indication and age, is *Mycophenolate Mophetile* (MMF). This is a prodrug of mycophenolic acid (MPA), an inhibitor of inosine-5’-monophosphate dehydrogenase (IMPDH). MPA depletes guanosine nucleotides preferentially in T and B-lymphocytes and inhibits their proliferation, thereby suppressing cell-mediated immune responses and antibody formation. Moreover, MPA can induce apoptosis of activated T-lymphocytes, which may eliminate clones of cells responding to antigenic stimulation (74).

In a single-centre experience, Miano et al. (25) reported that 65% (22/34) of children with AIC refractory to first line treatment showed a good response to MMF, especially in ALPS patients (11/11, 100%). This finding was confirmed in another single-centre study in USA (26) reporting an excellent response (12/13) to MMF in ALPS patients with refractory autoimmune cytopenia.

Although MMF dosage used in kidney transplanted patients is 1200 mg/m^2^/day in two doses, clinicians actually choose the minimum dose required to guarantee a good blood cell count and avoid the adverse events of chronic immunosuppression in PID patients.

Immunosuppression, in fact, increases susceptibility to bacterial, viral and fungal infections and the risk of development of lymphoma and other malignancies. Of note, baseline immunosuppression containing MMF regimen was recently reported as an independent predictor of severe COVID-19 thus suggesting a further serious reflection before its prescription during the current pandemic period (75).

### Targeting B cells

B-lymphocytes are responsible for autoantibodies production; thus, they represent an optimal specific target to stop autoimmunity.

*Rituximab* is a monoclonal antibody that targets CD20, a molecule constitutively and specifically expressed on B-cells surface. Its action is, indeed, to inhibit auto reactive B cells producing self-autoantibodies. According to different authors, Rituximab is considered an effective second or third line off-label treatment for AIC in PID, if administered at a dose of 375mg/m2 weekly for a total of three to four doses (27). In eight PID patients, responses occurred for 90% of treatments but relapse rates were high (78%) (28). Although the toxicity profile of rituximab is generally favourable, infusion reactions can rarely occur. Pre-treatment with acetaminophen and antihistamines is standard practice, rarely in association with hydrocortisone, when a previous history of severe reaction is reported. In some cases, Rituximab can induce prolonged absence of circulating B cells, especially memory B cells, and hypogammaglobulinemia requiring Ig replacement therapy. Chronic hypogammaglobulinemia has been reported in 32% of paediatric patients treated with Rituximab and 17% of them were eventually diagnosed with a PID (29).

For this reason, Rituximab is preferred in patients with CVID or other forms of PID already characterized by antibodies deficiency. In a cohort of 25 patients with CVID, treated for refractory cytopenia, Rituximab resulted more effective than other immunosuppressant drugs or immune modulators, with a high response rate (85%) (30). However, randomized clinical trials are still lacking and relapses have been observed. Rituximab treatment failure could be linked to the drug ability to deplete only maturing B cells while sparing antibody producing long-lived plasma cells (CD20-) that sustain autoimmune phenomena.

In line with this, other biological drugs that target B cells have been developed.

*Bortezomib* is a proteasome inhibitor that targets plasma cells (CD19+CD20−CD27^high^), causing their apoptosis *via* an augmented unfolded protein response and the induction of endoplasmic reticulum stress. It seems to have also other immunomodulatory properties like downregulation of inflammatory signalling conveyed by NF-kB, depletion of autoreactive T-helper cells, and interference with antigen processing and presentation. Bortezomib is approved for multiple myeloma treatment (31). Therefore, its use in AIC is off label for indication. Some clinical studies highlighted its efficacy in reducing autoantibodies production in several models of autoimmune conditions, including AIC (32, 33). It has been useful in treating AIC associated with immune dysregulation (34–37). In a small cohort of 13 patients with refractory AIC, 6 of which with underlying PID, Bortezomib showed an overall response rate of 77%, including 38% complete remissions (34). It has also been used as rescue therapy in a del22syndrome patient with ES refractory to second line therapies (38). Bortezomib is administered intravenously or subcutaneously at a starting dose of 1.3 mg/sqm on 21-day cycles (34).

*Daratumumab*, an anti-CD38 monoclonal antibody developed to target tumoral plasma cells in multiple myeloma (MM), induces the killing of CD38-bearing cells through different mechanisms of action (39). It has been shown to be effective in single case reports or case series of refractory AIHA and ITP (40–42) The main adverse event of this drug is hypogammaglobulinemia with risk of severe infections. In a study of 8 adult patients treated with Daratumumab for refractory AIC, 4 patients showed a good clinical response after four daratumumab infusions. Therefore, the risk/benefit balance of such therapy should be carefully discussed according to the patient’s clinical history. A multicentre phase II clinical trial is ongoing for the use of *Daratumumab* in ITP (NCT04703621) (43).

*Isatuximab*, a new drug targeting CD38 has recently been approved for the combination treatment of refractory MM (44). A clinical trial is ongoing for its use in warm AIHA (NCT04661033) (45).

*Belimumab*, a human mAb that inhibits B-cell-activating factor (BAFF) has been successfully used in the treatment of cytopenia in patients with systemic lupus erythematosus (SLE) (46).

A recent clinical trial showed a good clinical response in 15 splenectomised adults with chronic ITP using a combination regimen of rituximab plus five belimumab infusions at a dose of 10 mg/kg showing a higher decreasing of T follicular helper (Tfh) cells compared to patients receiving only rituximab (47). So it could be an option also in PID patients.

*Atacicept*, a fusion protein that inhibits both BAFF and anti-A proliferation-inducing ligand (APRIL), showed safety and efficacy in the treatment of flares in SLE (48), but has not been trialled in PID yet.

*Ibrutinib*, a small molecule inhibitor of Bruton’s tyrosine kinase (BTK), has been successfully used in the treatment of refractory AIHA among chronic lymphocytic leukaemia patients (49, 50) and could be an option to consider in PID.

Finally, anti-CD22 monoclonal antibody, *Epratuzumab*, has also being trialled in refractory cytopenias in patients with SLE (51) and could become another drug to use in PID associated cytopenias.

### Targeting T cells

Autoreactive T lymphocytes are key players in autoimmune diseases. They can act both as regulatory and effector cells, representing another optimal target for specific treatment.

*Sirolimus* (also known as Rapamycin) and *Everolimus* inactivate the serine/threonine protein kinase mechanistic target of rapamycin (mTOR), a key enzyme for the progression of cellular cycle (from G1 to S phase), thereby inhibiting cell growth and differentiation, in particular of peripheral T cells, but sparing regulatory T cells (Tregs), as shown in Immune-dysregulation, Polyendocrinopathy, Enteropathy, X-linked (IPEX) patients (52), thereby proving beneficial in the clinical setting of immune dysregulation. Sirolimus showed efficacy for the treatment of AIC as second line off-label therapy, especially in ALPS patients. A multicentre prospective clinical trial published in 2016 (53) has used Sirolimus as monotherapy (at a dose of 2 to 2.5 mg/m2 per day for 6 months) in adult and paediatric patients affected with multi-lineage AIC refractory to standard treatment with a complete remission achieved in all ALPS patients (12/12) and in most patient with CVID, ES, SLE, Activated PI3K-kinase Delta Syndrome (APDS) (8/12). Moreover, Sirolimus has been described as rescue therapy in patients with ITP who had previously failed to respond to MMF treatment (54).

It has been demonstrated that a hyperactive mTOR signalling promotes lymphoproliferation and high double negative T cells (DNT) production in ALPS patients (55). Rapamycin abrogates survival and proliferation of ALPS DNT cells, causing a significant reduction of lymphoproliferation in these patients. Sirolimus/Everolimus are used for many other PIDs, such as CVID, ES, CTLA4 def (56), ARPC1B deficiency, to control autoimmune phenomena.

*Abatacept (or Belatacept*), a fusion molecule composed by the extracellular domain of Cytotoxic lymphocyte antigen 4 (CTLA4) joined to the Fc-region of IgG1, is able to act as a CTLA4 agonist, inhibiting the hyperactivation of the immune response. Specifically, CTLA4 is a receptor on T cells that inhibits T cell activation and proliferation. Its ligands CD80 and CD86, are expressed by antigen-presenting cells (APCs) and the intracellular signal is mediated by PI3K-AKT-mTOR pathway.

CTLA4 haploinsufficiency is characterized by recurrent sinopulmonary infections, autoimmune manifestations and lymphoproliferation. AIC has been reported in 59% of these patients (56).

Thus, Abatacept represents a valid therapeutic option for treating AIC in CTLA4 haploinsufficiency with a high response rate (56, 57). LRBA is an intracytoplasmic protein that rescues CTLA4 from degradation allowing its recycling on the cell surface. The molecular interaction between LRBA and CTLA-4 has paved the way for the use of Abatacept even in this disease, with a good response (58).

Several PI3Kδ inhibitors have been trialled for the treatment of APDS patients. APDS, caused by heterozygous mutations in *PIK3CD* or *PIK3R1* genes (APDS1 and APDS2 respectively), is characterized by increased risk of multilineage cytopenias, lymphoproliferation especially B-cell lymphoma and recurrent infections. One of the first trialled drug has been *Lesiolisib* (59) that showed a good efficacy and tolerability in APDS1 patients. All patients had cytopenias at baseline and improved at the end of the 12-week treatment period.

Moreover, an open label phase 1b study on *Seletalisib* (60) demonstrated an acceptable safety profile and positive effects on several clinical features and disease activity in patients with APDS1 and APDS2.

### Targeting T cells and the inflammasome

Ongoing research suggest that the interplay of inflammasome mediators with immune modulators and transcription factors could have a significant role in the development of AIC and, possibly, in the resistance to standard therapies. Among pathways involved in immune dysregulation diseases JAK/STAT pathway could induce cytopenia trough different mechanisms and could be used, indeed, as a target for treatment.

Currently five different small molecules Jak inhibitors (Jakinibs) are available: *Tofacitinib* (JAK1 and JAK3 inhibitor), *Ruxolitinib* (JAK1 and JAK2 inhibitor), *Baricitinib* (JAK1 and JAK2 inhibitor), *Filgotinib* (a more selective JAK1 inhibitor), and *Decernotinib* (a selective JAK3 inhibitor). They are all off-label for AIC and in pediatric patients.

*Ruxolitinib* has been used to treat AIC in patients affected by gain-of-function (GOF) *STAT1* and GOF *STAT3* mutation, showing a good response (61). It reduces IFNγ activity, normalizes T_H_1 and T_FH_ cell differentiation, and improves T_H_17 cell development, managing an effective control of immune-dysregulation (62).

Jak inhibitors could represent valid therapeutic options in diseases involving the JAK/STAT pathway (63, 64).

*Anakinra* is a recombinant version of interleukin-1 receptor antagonist approved for the treatment of rheumatoid arthritis. Nowadays it is used for many other inflammatory diseases, like MIS-C (multisystem inflammatory syndrome in children) (66, 67), secondary HLH and to treat cytopenia in Wiskott Aldrich Syndrome (WAS) patients before going to gene therapy/HSCT (65).

Tumor necrosis factor (TNF)-alpha is a potent pro-inflammatory and pathological cytokine in inflammatory diseases. TNF inhibitors, including etanercept, adalimumab, infliximab, and golimumab, are used in several autoinflammatory disorders, including the IEI deficiency of adenosine deaminase 2 (DADA2), in which cytopenias, either autoimmune or due to bone marrow failure (BMF), occur in 50% of patients. Actually, the efficacy of TNF inhibitors on cytopenia is temporary and poor. In a study of 31 DADA2 patients (68), TNF inhibitors significantly reduced the incidence of ischemic events and other vasculitic manifestations but was not effective for immunodeficiency or bone marrow failure, requiring a HSCT.

### Other therapeutic approaches

Eventually, in some PIDs the pathogenic mechanism of cytopenia is not purely autoimmune.

Indeed, in most of cytoskeletal disorders such as WAS, DOCK8 deficiency, CDC42 deficiency and ARPC1B deficiency, cytopenia result from an intrinsic cellular defect and splenic removal of abnormal cells. In addition, an immune mediated mechanism could be involved (76).

In these diseases steroids usually work but other drugs are often needed.

*Eltrombopag* (ELT) and *Romiplostin* are thrombopoietin receptor (TPO-R) agonists approved in chronic ITP for stimulating platelet production (10, 13). They also have immunomodulating properties by stimulating T and B regulatory cell activity and by promoting a macrophage switch from the pro-inflammatory to the anti-inflammatory phenotype. They are used in WAS patients to help platelet production, in addition to other immunomodulating therapies already discussed.

In WHIM syndrome (Warts, hypogammaglobulinemia, infections, and myelokathexis) due to *CXCR4* gain of function mutation, neutropenia is caused by accumulation of mature and degenerating neutrophils in the bone marrow, whereas AIHA and/or ITP can occur.

*Plerixafor* is a CXCR4 subcutaneous inhibitor known to be used for stem cell mobilization in cancer. It has been recently shown to be effective in WHIM syndrome, with the resolution of neutropenia, anemia, and thrombocytopenia (69, 70). It acts increasing the mobilization of neutrophils from the bone marrow restoring peripheral levels. A clinical trial with *Mavorixafor*, an oral drug, is currently in progress (71).

The complement system plays an important role in the physiopathology of AIHA. Autoantibodies, indeed, can activate the complement system *in vivo*, leading to the destruction of red blood cells. The terminal complement inhibitor *Eculizumab* (anti-C5) has been used as rescue therapy of AIHA in a patient with Waldenström macroglobulinemia (72) and in a patient with chronic myelomonocytic leukemia (CMML) (73). Its cost, the suspensive effect and the risk of severe bacterial infections such as meningococcal infection could represent his main limitation. It has never been trailled in PID yet.

## HSCT/GT

The indication to Hematopoietic Stem Cell Transplantation (HSCT) or Gene Therapy (GT) is strictly correlated to the type of PID, when diagnosed, and to a severe clinical and immunological picture and cytopenia refractoriness.

HSCT is the gold standard definitive therapy for many specific PIDs, especially in childhood (77). It is mandatory and urgent for all SCIDs and most of CIDs, while in Primary Immune dysregulation disorders (PIRDs) and other less profound IEI, the decision in favour or against HSCT is often challenging since a conservative therapy is possible, but progressive organ damage and severe inflammation could worsen HSCT prognosis (77). HSCT was an effective treatment for DADA2, in which cytopenia is generally related to bone marrow failure than to immune mediated mechanism, successfully reversing the refractory cytopenia in a cohort of 30 DADA2 patients (78).

Indeed, HSCT should be discussed case by case and targeted agents could represent a bridging therapy.

GT has been explored for a limited number of IEI, including ADA-SCID, X-linked SCID, XL-CGD, and WAS. A retroviral ADA GT product (Strimvelis^®^) is licensed by the European Medicines Agency (EMA), and there are ongoing clinical studies in a variety of other IEI (79). Recently GT in WAS showed good outcome and long-term follow-up (80).

## Conclusions

The clinical course of AICs in PID patients could be really challenging. Haematologists and immunologists should jointly care for this selected patient cohort from the beginning of treatment and onward. The progress in molecular studies has allowed the development of many novel therapeutic agents that could be useful for a targeted treatment of refractory AICs in PID patients. For some PIDs, a targeted therapy can compensate for the underlying defect, restoring the impaired signalling pathway and potentially correcting the accompanying AIC. Actually, the molecular diagnosis or a specific drug is not always available. However, defining the category of PID or the immunological phenotype can help to choose a second-line therapy that is at least directed towards the suspected pathomechanism. HSCT/GT should be considered basing on the type of PID and the cytopenia refractoriness to any line of therapy. To support decision making in the future, prospective studies to define treatment response–predicting or –stratifying biomarkers for patients with AIC and PID are needed.

## Author contributions

LP, GG, AF and CaC conceptualized the review idea. LP did the bibliography research and wrote the manuscript. PP, AF and CaC supervised the literature research and the manuscript redaction. GG, DA, BR, GAR, EM, GP and CC contributed to the bibliography research and to a critical revision of the manuscript. PR and CP supervised the manuscript revision. All the authors have read and approved the manuscript.

## Funding

The study was supported by grants of the Ricerca Corrente 5x1000 from Childrens’ Hospital Bambino Gesù, Rome, Italy (2021105_INFETT_CANCRINI).

## Conflict of interest

The authors declare that the research was conducted in the absence of any commercial or financial relationships that could be construed as a potential conflict of interest.

## Publisher’s note

All claims expressed in this article are solely those of the authors and do not necessarily represent those of their affiliated organizations, or those of the publisher, the editors and the reviewers. Any product that may be evaluated in this article, or claim that may be made by its manufacturer, is not guaranteed or endorsed by the publisher.
